# COVID-19 misinformation on YouTube: An analysis of its impact and subsequent online information searches for verification

**DOI:** 10.1177/20552076231177131

**Published:** 2023-05-25

**Authors:** Sabrina Heike Kessler, Edda Humprecht

**Affiliations:** 1 Department of Communication and Media Research, University of Zürich, Zürich, Switzerland; 2 Department of Sociology and Political Science, 8018 Norwegian University of Science and Technology (NTNU), Trondheim, Norway

**Keywords:** Misinformation, YouTube, COVID-19 vaccination, internet search behavior, vaccination attitudes, eye tracking

## Abstract

**Objectives:**

COVID-19 vaccination misinformation on YouTube can have negative effects on users. Some, after being exposed to such misinformation, may search online for information that either debunks or confirms it. This study's objective is to examine the impact of YouTube videos spreading misinformation about COVID-19 vaccination and the influencing variables, as well as subsequent information seeking and its effect on attitudes toward vaccination.

**Methods:**

In this observational and survey study, we used a three-group pre-test and post-tests design (*N* = 106 participants). We examined the effects of YouTube videos containing misinformation about COVID-19 vaccination on attitudes toward vaccination via surveys, employed screen recordings with integrated eye tracks to examine subsequent online information searches, and again surveyed participants to examine the effects of the individual searches on their attitudes.

**Results:**

Receiving misinformation via video tended to have negative effects, mostly on unvaccinated participants. After watching the video, they believed and trusted less in the effectiveness of the vaccines. Internet searches led to more positive attitudes toward vaccination, regardless of vaccination status or prior beliefs. The valences of search words entered and search duration were independent of the participants’ prior attitudes. Misinforming content was rarely selected and perceived (read). In general, participants were more likely to perceive supportive and mostly neutral information about vaccination.

**Conclusion:**

Misinformation about COVID-19 vaccination on YouTube can have a negative impact on recipients. Unvaccinated citizens in particular are a vulnerable group to online misinformation; therefore, it is important to take action against misinformation on YouTube. One approach could be to motivate users to verify online content by doing their own information search on the internet, which led to positive results in the study.

## Introduction

Worldwide, millions of people have been negatively affected by the COVID-19 pandemic.^1^ Success in reducing that burden depends on public awareness and acceptance of health interventions and, in particular vaccination rates. During the pandemic, people search the internet for information about COVID-19 vaccination to form their own opinions about the vaccine, which may influence their pandemic response.^2^ The pandemic caused an overabundance of information, especially online, as well as misinformation and disinformation—known as an *infodemic*.^3^ Health-related misinformation has had serious socioeconomic consequences; misinformation that promoted vaccine hesitancy resulted in a higher number of COVID-19 cases, hospitalizations, deaths, and millions in additional healthcare costs.^4^

Misinformation is false information that is disseminated regardless of the intent to mislead.^5^ That is, valid (scientific) evidence indicates that the information in question is false. This includes disinformation, which is the intentional dissemination of misinformation.^5^ Misinformation is spread primarily through social media, such as YouTube.^6^ YouTube is a frequently used source of COVID-19 information.^7^ However, the effects of misinforming content on YouTube on recipients’ attitudes and variables influencing the effect are poorly researched. We know from general survey studies that belief in misinformation can have a variety of negative effects and makes pandemic control more difficult.^4,8–12^

After users encounter misinformation on the internet, one way to deal with it is to check it for accuracy before believing it. Verifying suspicious information is a type of information search that aims to form a personal opinion about authenticity and accuracy based on additional information.^13^ Very little research has investigated what such information searches look like specifically, and how searches might then influence attitudes and credibility attributions toward the misinforming content.

In this study, we use a multimethod design to examine the impact of misinforming YouTube videos on COVID-19 vaccination and influencing variables, as well as subsequent information seeking and its effect on recipients’ attitudes toward vaccination. A better understanding of these impact and influence processes will allow us to identify risk factors and recommend targeted interventions against the belief in and spread of misinformation.

### Misinformation on YouTube: dissemination and impact

YouTube is a source of information on COVID-19 that is used regularly in many countries.^14^ In countries with greater distrust of government-appointed experts, trust in information about COVID-19 is greater on social media.^7^ People with less trust in public instances and who consume less traditional news media on COVID-19 are more likely to believe misinformation on measures against it.^15^

A recent study shows that when participants felt they could not obtain trustworthy information on COVID-19 from established channels, they turned to online sources, such as YouTube.^6^ However, YouTube has been criticized for distributing misinforming content and has been a significant source of misleading information during former public health crises, including the H1N1 (swine flu), Ebola, and Zika outbreaks.^16–19^ Misinforming videos related to COVID-19 reached high numbers of viewers.^6,20^ According to a survey conducted by Allington et al.^21^ in the UK, YouTube was the information source most strongly associated with belief in conspiracy theories. Studies reported that approximately one-quarter of the most viewed or automatically recommended YouTube videos were misleading, whereas videos from reputable sources were vastly underrepresented.^16–18^ Those studies examining the extent of COVID-19 misinformation indicated that it is reaching more individuals than in past public health crises, as YouTube continues to grow as a source of health information. The frequency of misinforming COVID-19 videos is 10%–26% depending on the study, time of measurement in the pandemic, and research design.^20,22,23^ Notably, misinformation about COVID-19 vaccination has also spread, which is seen as particularly problematic for pandemic containment because it may discourage recipients from vaccination.^24,25^

Numerous studies worldwide suggest that belief in COVID-19 misinformation is associated with decreased willingness to undertake effective prevention measures, like taking COVID-19 diagnostic or antibodies tests, adherence to social distancing guidelines and using the contact tracing application, and lower vaccination intent.^4,8–12^ The belief in mis- and disinformation poses major challenges for effective health communication and can hinder pandemic containment.

Usually, misinformation does not directly affect recipients’ attitudes and behavior. We know from impact research that prior attitudes and past behavior in particular can influence how and whether information/media content affects attitudes.^26,27^ One explanation for this finding is offered by Festinger's theory of cognitive dissonance, which assumes that individuals strive to establish consistency in their cognitions and between their cognitions and behavior.^28^ Cognitive dissonance is assumed to be a state of mental imbalance that feels emotionally uncomfortable, which immediately motivates individuals to overcome this state and achieve consonance once again.^28^ Contrary arguments or influencing attempts actually can reinforce existing attitudes, such as when individuals actively search for confirming arguments (cf. also worldview backfire effect).^25,29^ Moreover, prior attitudes have been repeatedly demonstrated as consequential to beliefs in misinformation and the effectiveness of corrective interventions.^30,31^ In media effects research, the attributed credibility of a message and a source are also regarded as decisive influencing variables.^32,33^ The attributed credibility also affects, for example, the effect of debunking texts against health-related misinformation.^34^

The theory of cognitive dissonance is not the only explanatory factor at play here; there are other close allies. For example, when people who hold strong beliefs are faced with arguments challenging those beliefs, they are prone to skepticism (and disbelief, as opposed to discomfort) and will engage in disconfirmation bias, or extended search (through their memory, in the case of Edwards & Smith) for refutational arguments.^35,36^

Building on the research discussed above, the main research question (RQ1) of this study relates to whether recipients’ attitudes toward COVID-19 vaccination differ before and after watching a vaccination-critical misinforming video.

RQ1: What is the impact of a misinforming YouTube video about COVID-19 vaccination on recipients’ attitudes toward vaccination?

Past behavior and prior attitudes can influence how information/media content affects attitudes.^26,27^ The effect of a misinforming video critical of vaccination, according to the theory of cognitive dissonance and disconfirmation bias, is likely to be different for people who have decided to get vaccinated than for people who have not been vaccinated.^28,35^ It is likely that the negative preconceptions of unvaccinated individuals are reinforced, and vaccinated individuals are more likely to suppress the dissonant content.

H1: Depending on the vaccination status (H1a) and prior attitudes (H1b) of the recipients, the effect of the misinforming videos differs.

### Online information behavior and selective exposure

Digitalization has changed the way individuals search for health information, and digital technologies provide many people with easy access to health information in general and on COVID-19 and its vaccine in particular.^2^ Surveys have examined how people obtain information on health topics such as COVID-19.^14,37–39^ The internet is generally one of the most important sources for information on health and specifically COVID-19.^14,15^ Psychological drivers of information seeking concerning COVID-19 include emotional appraisals, such as fear, and the perceptions of risk and uncertainty.^40^ Unfortunately, few people verify the (mis-)information they find on the internet.^13,41^ Sun examined the factors that motivate people to verify health misinformation found on social media and the outcomes of their efforts.^13^ The study showed that individuals with higher levels of fear were more likely to engage in institutional verification by using search engines, reputable medical websites, or fact-checking sites. Intention to engage in institutional verification increased their belief in the efficacy of correcting misinformation, which motivated them to do so on social media. Studies show that online information seeking about COVID-19 had a positive impact on intention to engage in preventive behaviors, such as wearing masks or vaccination.^2,42^

However, online vaccination information overload can trigger internal states that may reduce vaccination intention.^2^ Therefore, individuals need sufficient skills to search systematically for relevant information. In this context, the demand for increased digital health literacy has been voiced.^37,43^ Few observational studies exist that examine topic-related online information behavior on an individual level. Those studies show that health-related information reception and search behavior are usually stable and habitualized, as is search behavior in general.^44,45^ For example, search engines’ rankings play an important role in viewing behavior and information selection resulting in more frequent access to big, general, and moderate websites instead of small, extreme, or very specific offerings.^44–46^ The assumed quality of information and accessibility to information channels also play a role in information search.^47^

The advanced options online make it easier for users to search selectively. Users are therefore increasingly able to select information that correspond to their attitudes. According to research on selective exposure, individuals consciously or unconsciously select media offerings that correspond to their own attitudes because they strive to establish consistency between their cognitions.^48^ Selective exposure research is partly based in part on Festinger's theory.^28^ Several experimental studies confirm the assumption of selective exposure with respect to online information search.^49–51^ However, these studies usually use self-created mock websites and time limits. Studies with a more real-world experimental design tend not to confirm a primarily attitude-directed information search.^45,52^ When people are confronted with misinformation, however, different results emerge. Exposure to attitude-contrary misinformation can trigger individuals’ additional information seeking to verify the information that they suspect to be false.^53^ The multimethod study by Wolff and Taddicken suggests that users often attempt to resolve cognitive dissonance triggered by misinformation through confirmatory online information seeking.^54^

The study's second RQ deals with verification and falsification processes after exposure to misinforming videos.

RQ2: How do people inform themselves online after watching misinforming YouTube videos about COVID-19 vaccination?

According to the selective exposure theory, it can be assumed that recipients’ attitudes affect the information search and that, accordingly, they are more likely to search for and perceive (read) attitude-consonant content.^48^

H2: When searching for information online, participants are more likely to search for and read attitude-consonant than dissonant content.

According to the theory of disconfirmation bias, one prediction is that participants with more positive attitudes toward the COVID-19 vaccination will spend more time searching the internet and specifically looking for information that refutes the misinformation after being confronted with a misinforming video.^35^

H3: People with more positive attitudes toward COVID-19 vaccination will spend more time searching the internet after being confronted with a misinforming video and specifically seeking information that refutes the misinformation than people with negative attitudes.

The last RQ asks about the effect of online information searches on recipients’ attitudes toward COVID-19 vaccination. It is examined whether the searched and received content contributes to debunking the received misinformation or increasing belief in it.

RQ3: What is the impact of online information searches on recipients’ attitudes toward COVID-19 vaccination?

A simple online search can lead to uncovering the obvious misinformation in the videos, so the credibility attributed to them after reception should decrease after the search.

H4: The online information search decreases recipients’ credibility attribution to the misinforming videos.

## Methods

Based on the findings of a content analysis from Humprecht and Kessler, we selected three prototypical videos as stimulus material.^55^ The quantitative content analysis of misinforming German- and French-speaking YouTube videos (*n* = 450) examined types, statements, actors, and attributions of responsibility which can be found in misinformation about COVID-19. Criteria for selection were that the videos belonged to one of the common types of misinformation videos, the language of the videos, the presence of mainly partially false information, a similar length of the videos of over 10 and under 15 minutes, recency of the video (not older than six months), and as the main topic of the video, vaccination against COVID-19. The three videos that were selected from the sample corresponded to the identified common types of misinformation videos: interview format, prominent vaccination opponent, and alternative media. The video in interview format showed an interview situation between three people, with one moderator interviewing two other men. One of them misinformatively blames the COVID-19 vaccination for the death of his father and the other speaks of a conspiracy regarding the vaccination. The video from the prominent vaccination opponent shows him misinterpreting study results on COVID-19 vaccination. The video from the alternative media outlet shows all sorts of misinformation about Bill Gates and his role in the COVID-19 vaccination. All three videos contain mainly partially false information as misinformation according to Hameleers et al.^56^ Facts are taken out of context and misinterpreted and the content stays close to reality but places facts outside of their context to alter their meaning. All of the videos were on YouTube, were not older than 6 months, were in German, and were similar in length at 11–12 minutes.

Each video was presented to *n* = 35–36 individual persons in the eye-tracking lab of the university of the first author. The sample size (*N* = 106) was calculated using a priori power analysis. We calculated the required sample size of 106 participants via G-Power (www.gpower.hhu.de). We wanted to be able to identify at least small effects (*f* = .20) in an ANOVA with repeated measures (within factors) with three groups and three repeated measures (with max α-error = .05 and power [1−ß-error] = .95) and also in two repeated measures (regarding the attributed credibility to the video after reception and after internet research). In addition, we wanted to calculate the effects of video reception on search behavior and the effects of search behavior on attitudes, respectively, using regression analysis, and to once again be able to identify at least small effects (*f* = .20; the number of predictors = 5; max α-error = .05 and power [1−ß-error] = .95).

The groups were representative in terms of gender, age, and educational level in relation to the Swiss population aged 18 and older.^57^ Participants were recruited via flyers and the university's test person pool and received 40 Swiss francs (about 42 USD) expense allowance (including travel costs). The study was conducted in November 2021, when infection and death rates in Switzerland were very high and still rising rapidly, and the government decided to take further actions to contain the pandemic.

**Figure 1.**
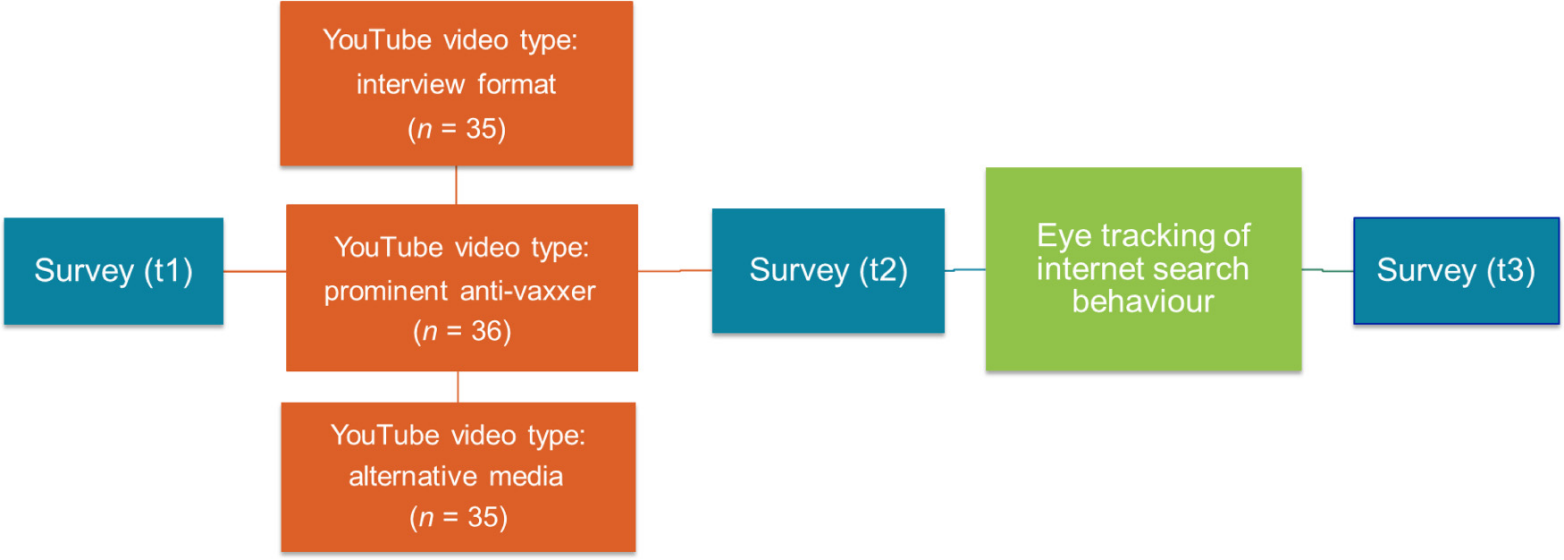
Design of the eye-tracking study.

Before the study began, an informed consent was obtained from all participants before study participation via printed and signed information sheet and consent forms. The study began with a standardized online presurvey (t1) on the laboratory computer (see Figure 1), which asked about general attitudes toward vaccination, YouTube usage and assessment of information about COVID-19 on YouTube, and self-assessed internet search ability and frequency (see Table 1 for question wording, items, scale levels, and means). Next, participants were placed in front of a stationary eye tracker. Data collection began after an initial calibration phase. In the calibration phase, the correct focus of the participants’ eyes was measured at nine points. For each participant, the data were within an acceptable range (derivation *x*: *M* = 0.58; *SD* = 0.20; derivation *y*: *M* = 0.51; *SD* = 0.24). After calibration, the participants were asked to watch one of the three randomly assigned videos. Afterward, participants were asked in a standardized online survey (t2) about their credibility attribution to the videos (content and speaker credibility according to Roberts), perceived need for further online information search, and attitudes toward COVID-19 vaccination (five items, including cognition, emotion, and behavioral intention; see Table 2 for wordings, items, scale levels, and means).^58^ Participants were asked to find evidence as to whether the content shown corresponded to the truth: “Please now inform yourself on the internet about the content of the YouTube video you have seen.” In the original wording and language: “Bitte informieren Sie sich nun im Internet über die Inhalte des gesehenen YouTube-Videos.” Participants were allowed to search as long as they liked and to open any website they wanted. Their online searches were recorded by the stationary eye-tracking device (SensoMotoric Instruments stationary remote system; iView X Red 120 Hz). We opted for this approach because analyzing online search behavior without external time constraints and in a real online environment leads to higher external validity than approaches used in most studies on online search behavior.^45^ In a standardized follow-up survey (t3), we asked again about participants’ credibility attribution to the video and attitudes toward COVID-19 vaccination, whether the video content was supported through the online search, how the search resembled participants’ everyday online search, trust in social actors regarding statements about COVID-19, self-assessed level of knowledge about COVID-19 vaccination, self-assessed internet skills, previous exposure to misinformation, individual behavior when dealing with misinformation, and experiences with the disease (see Table 3 for question wording, items, scale levels, and means). Participants were extensively debriefed in writing and verbally after the post-survey (t3). We used articles from the fact-checker correctiv.org and evidence-based information from the Swiss Federal Office of Public Health. The study was approved by the ethics committee of the first author's university.

**Table 1.** Survey (t1) constructs/variables, exact question wordings, items, scale levels, and means.

Construct	Question	Measurement details	Measurement items & characteristics	*Mean and standard deviation*
Attitudes toward COVID-19 vaccination	A. What is your attitude toward vaccination in general? B. Please mark how strongly you agree with the following statements.	A. Five-point semantic scale B. Five items; five-point Likert scale: 1 = *do not agree at all* to 5 = *agree*; items randomized	A. Totally positive–totally negative B. • I believe that vaccination against COVID-19 helps against severe infection. • I have advised other people to get vaccinated against COVID-19 before. • I am skeptical about the effectiveness of the COVID-19 vaccine. • I have confidence in the effectiveness of the COVID-19 vaccine. • I am afraid of (long-term) side effects of the COVID-19 vaccine.	A. *M* = 1.9; *SD* = 1.0 B. *M*_believe_ = 4.1; *SD* = 1.2 *M_advice_* = 3.8; *SD* = 1.5 *M_skepticism_* = 2.2; *SD* = 1.2 *M_confidence_* = 3.8; *SD* = 1.3 *M_fear_* = 2.2; *SD* = 1.3
COVID-19 vaccination	A. Are you fully vaccinated against COVID-19? B. FILTER: Do you still plan to get (fully) vaccinated against COVID-19? C. FILTER: Why not?	A. Nominal scale B. Nominal scale C. String	A. 1 = yes; 2 = no B. 1 = yes; 2 = no C. in words	A. *M_fully vaccinated_* = 1.1; *SD* = 0.3 B. *M_get vaccinated_* = 1.9; *SD* = 0.4
YouTube usage	A. How often do you use YouTube to get information? B. How often do you use YouTube for personal entertainment?	A. Five-point Likert scale B. Five-point Likert scale	Never–very often	A. *M_YT Information_* = 3.2; *SD* = 1.2 B. *M_YT entertainment_* = 4.1; *SD* = 1.1
Information about COVID-19 on YouTube	I rate the information about the COVID-19 vaccination on YouTube as…	Adjusted according to Science Barometer Switzerland, 2020; 10 items; five-point Likert scale: 1 = *do not agree at all* to 5 = *agree*; don’t know option; items randomized	• Trustworthy • Understandable • Detailed • Correct • Exaggerated • Annoying • Independent • Well researched • High quality • Helpful	*M_trustworthy_* = 2.7; *SD* = 0.9 *M_understandable_* = 3.6; *SD* = 0.9 *M_detailed_* = 3.1; *SD* = 1.1 *M_correct_* = 2.6; *SD* = 0.9 *M_exaggerated_* = 3.5; *SD* = 1.0 *M_annoying_* = 2.9; *SD* = 1.1 *M_independent_* = 2.6; *SD* = 1.0 *M_researched_* = 2.7; *SD* = 0.9 *M_high quality_* = 2.7; *SD* = 0.9 *M_helpful_* = 3.2; *SD* = 1.0
Internet search ability	How would you rate your ability to search specific topics on the Internet?	One item; five-point Likert scale	Very high–very low	*M* = 1.9; *SD* = 0.9
Internet search frequency	How often do you search for information on the Internet?	Five-point semantic differential scale	1 = hourly; 2 = daily; 3 = weekly; 4 = monthly; 5 = never	*M* = 1.6; *SD* = 0.7
Gender	Which gender do you feel you belong to?	Nominal scale 1–3	1 = female; 2 = male; 3 = diverse	
Education	What was the last level of education you completed?	Nominal scale 0–3	0 = no education completed; 1 = compulsory school; 2 = secondary education; 3 = tertiary education	
Age	How old are you?	0–*x*	18–*x*	*M* = 36.4; *SD* = 17.4

*Note.N* = 105.

**Table 2.** Survey (t2) constructs/variables, exact question wordings, items, scale levels, and means.

Construct	Question	Measurement details	Measurement items & characteristics	*Mean and standard deviation*
Credibility of the content/speaker	A. How do you rate the content of the video you just watched? B. How do you rate the speaker of the video you just watched?	A. Credibility of content (Roberts, 2010); five items; semantic differential scale; items randomized B. Credibility of speaker (Roberts, 2010); five items; semantic differential scale; items randomized	A. • Biased–unbiased • Unbelievable–believable • Trustworthy–not trustworthy • Inaccurate–accurate • Incomplete–complete B. • Can be trusted–cannot be trusted • Fair–unfair • Inaccurate–accurate • Complete (tells the whole story)–incomplete (does not tell the whole story) • Unbiased–biased	A. *M_bias_* = 2.2; *SD* = 1.1 *M_belief_* = 2.5; *SD* = 1.2 *M_trustworthy_* = 3.8; *SD* = 1.1 *M_exact_* = 2.4; *SD* = 1.1 *M_accurate_* = 1.9; *SD* = 1.0 B. *M_trustworthy_* = 3.8; *SD* = 1.1 *M_fair_* = 3.2; *SD* = 1.0 *M_accurate_* = 2.3; *SD* = 1.1 *M_complete_* = 4.1; *SD* = 1.1 *M_bias_* = 4.1; *SD* = 1.1
Need to inform about content	How highly do you rate your desire to now find out about the video content on the Internet?	Seven-point semantic differential scale	Strong–weak	*M* = 4.3; *SD* = 1.9
Attitudes about COVID-19 vaccination	Please also mark how strongly you agree with the following statements.	Five items; five-point Likert scale: 1 = *do not agree at all* to 5 = *agree*; don’t know option; items randomized	• I believe that vaccination against COVID-19 helps against severe infection. • I have advised other people to get vaccinated against COVID-19 before. • I am skeptical about the effectiveness of the COVID-19 vaccine. • I have confidence in the effectiveness of the COVID-19 vaccine. • I am afraid of (long-term) side effects of the COVID-19 vaccine.	*M_believe_* = 4.3; *SD* = 1.1 *M_advice_* = 3.9; *SD* = 1.5 *M_skepticism_* = 2.2; *SD* = 1.3 *M_confidence_* = 3.8; *SD* = 1.3 *M_fear_* = 2.3; *SD* = 1.3

*Note.N* = 105.

**Table 3.** Survey (t3) constructs/variables, exact question wordings, items, scale levels, and means.

Construct	Question	Measurement details	Measurement items & characteristics	*Mean and standard deviation*	
Assessment of internet search	A. Were you able to confirm the video content you saw by means of your Internet search? B. Did the Internet search behavior you just performed match your normal/everyday online search behavior?	A. Five-point semantic differential scale B. Five-point semantic differential scale	Yes–no	A. *M* = 3.6; *SD* = 1.3 B. *M* = 2.1; *SD* = 0.8	
Assessment of video content	A. How do you rate the content of the video you just watched? B. How do you rate the speaker of the video you just watched?	A. Credibility of content (Roberts, 2010); five items; five points; semantic differential scale; items randomized B. Credibility of speaker (Roberts, 2010); five items; five points; semantic differential scale; items randomized	A. • Biased–unbiased • Unbelievable–believable • Trustworthy–not trustworthy • Inaccurate–accurate • Incomplete–complete B • Can be trusted–cannot be trusted • Fair–unfair • Inaccurate–accurate • Complete (tells the whole story)–incomplete (does not tell the whole story) • Unbiased–biased	A. *M_bias_* = 1.9; *SD* = 1.2 *M_belief_* = 2.1; *SD* = 1.2 *M_trust_* = 4.2; *SD* = 1.0 *M_acc_* = 2.0; *SD* = 1.2 *M_comp_* = 1.8; *SD* = 1.1 B. *M_trust_* = 4.1; *SD* = 1.2 *M_fairness_* = 3.7; *SD* = 1.0 *M_acc_*= 2.0; *SD* = 1.1 *M_comp_* = 4.3; *SD* = 0.9 *M_bias_* = 4.3; *SD* = 1.0	
Search behavior on various topics about COVID vaccination	Prior to this study, have you ever… • searched for specific content from vaccination critics on the internet? • searched the internet for information about vaccination against COVID-19?	Five-point semantic differential scale	No never–very often	*M_vaccritics_* = 2.4; *SD* = 1.3 *M_vacinfo_* = 4.0; *SD* = 1.2	
Attitudes about COVID-19 vaccination	Please also mark how strongly you agree with the following statements.	Six items; five-point Likert scale: 1 = *do not agree at all* to 5 = *agree*; don’t know option; items randomized	• I believe that vaccination against COVID-19 helps against severe infection. • I have advised other people to get vaccinated against COVID-19 before. • I am skeptical about the effectiveness of the COVID-19 vaccine. • I have confidence in the effectiveness of the COVID-19 vaccine. • I am afraid of (long-term) side effects of the COVID-19 vaccine.	*M_belief_* = 4.3; *SD* = 1.0 *M_advice_* = 3.8; *SD* = 1.5 *M_skepticism_* = 2.1; *SD* = 1.3 *M_confidence_* = 4.0; *SD* = 1.2 *M_fear_* = 2.3; *SD* = 1.3	
Trust	Now please mark how much you trust statements about COVID-19 from the following actors:	Science Barometer Switzerland, 2020; six items; five-point Likert scale: 1 = *do not agree at all* to 5 = *agree*; don’t know option; items randomized	• Scientists and researchers • Physicians and medical staff • Politicians • Representatives of cantonal authorities and federal offices • Journalists • Relatives, acquaintances, colleagues	*M_scientists_* = 4.5; *SD* = 0.9 *M_physicians_* = 4.2; *SD* = 1.0 *M_politicians_* = 2.5; *SD* = 1.0 *M_represent_* = 3.4; *SD* = 1.1 *M_journalists_* = 2.7; *SD* = 1.0 *M_relatives_* = 2.6; *SD* = 0.9	
Knowledge assessment	Please indicate your opinion on the following statements.	Knowledge assessment (Science Barometer Switzerland, 2020); four items; seven-point Likert scale: 1 = *do not agree at all* to 7 = *agree*; don’t know option; items randomized	• I know a lot about vaccination against COVID-19. • I feel unsettled by the extensive, partly contradictory, information about vaccination against COVID-19 from different sources. • I follow the media coverage of COVID-19 very closely. • Compared to most others, I know less about vaccination against COVID-19.	*M_knowledge_* = 4.5; *SD* = 1.5 *M_feeling_* = 3.4; *SD* = 2.0 *M_coverage_* = 4.2; *SD* = 1.9 *M_knowl_*= 2.6; *SD* = 1.3	
Internet skills	Please assess to what extent the following statements apply to you.	Internet skills (van Deursen et al., 2016); nine items; five-point Likert scale: 1 = *does not apply to me at all* to 5 = *strongly applies to me*; don’t understand option; items randomized	• I get tired when I search for information online. • I should take a course on how to find information online. • The different designs of websites make it difficult for me to find information online. • I find it difficult to decide which keywords to use for an online search. • I find it difficult to find a previously visited website again. • I sometimes find it difficult to review information I have retrieved. • Sometimes, I get to websites without knowing how I got there in the first place. • I find the design of many websites confusing. • I find it easy to distinguish between what is true and what is false when I am reading up on current news.	*M_search_* = 2.8; *SD* = 1.3 *M_find_* = 1.6; *SD* = 0.8 *M_designs_* = 2.4; *SD* = 1.3 *M*_keywords_ = 2.2; *SD* = 1.1 *M*_previous_ = 1.8; *SD* = 0.9 *M*_review_ = 3.0; *SD* = 1.2 *M*_random_= 2.4; *SD* = 1.4 *M*_design_ = 2.5; *SD* = 1.3 *M_truefalse_* = 3.6; *SD* = 1.1	
Misinformation	A Misinformation (fake news) about the vaccination against COVID-19 I encountered myself so far… B Now, thinking about your personal interaction with information in traditional media or on the Internet, how do you typically ensure that the information you encounter is accurate? C How do you respond when you come across news or information about COVID-19 vaccination that you suspect to be false?	A Three items; six-point semantic differential scale (1 = hourly, 2 = daily, 3 = weekly, 4 = monthly, 5 = semiannually, 6 = never yet); items randomized B Eight items; five-point Likert scale: 1 = *do not agree at all* to 5 = *agree*; don’t know option; items randomized C Four items; seven-point Likert scale: 1 = do not agree at all –7 = agree at all; don’t know option; items randomized	A • In general • On the internet • On YouTube B • I rely on journalists or news source. • I search for the original source of the information. • I compare different news sources to check the facts. • I check fact-checking websites to verify the information. • I talk about it with friends and peers. • I look up what other people are saying online (e.g. blogs, social media, comment columns) about the information. • I rely on my own knowledge of the topic. • I rely on my gut feeling. C • I ignore this information. • I point out to others that this information may be wrong. • I don’t respond to it, but I appreciate it when others correct the information. • I am annoyed by such information.	A. *M_igeneral_* = 3.1; *SD* = 0.9 *M_nternet_* = 3.1; *SD* = 1.1 *M_YouTube_* = 4.2; *SD* = 1.4 B. *M_rely_* = 2.9; *SD* = 1.1 *M_search_* = 4.1; *SD* = 1.0 *M_compare_* = 4.1; *SD* = 1.0 *M_check_* = 2.8; *SD* = 1.3 *M_talk_* = 3.6; *SD* = 1.1 *M_look_* = 2.9; *SD* = 1.4 *M*_knowl_ = 3.5; *SD* = 1.0 *M*_gut_ = 3.0; *SD* = 1.1 C. *M_ignore_* = 3.9; *SD* = 1.2 *M_point out_* = 3.2; *SD* = 1.2 *M_respond_* = 3.7; *SD* = 1.0 *M_annoyed_* = 4.0; *SD* = 1.1	
Personal involvement	A. Have you been infected with COVID-19? B. If yes: Was the course mild or severe? C. Have there been or are there people in your family or acquaintances who have been infected with COVID-19? D. Do you belong to a COVID-19 risk group?	A. Nominal scale B. Nominal scale C. Nominal scale D. Nominal scale	A. 1 = yes; 2 = no B. 1 = mild; 2 = severe C. 1 = yes; 2 = no D. 1 = yes; 2 = no	A. *M* = 1.9; *SD* = 0.3 B. *M* = 0.1; *SD* = 0.3 C. *M* = 1.2; *SD* = 0.4 D. *M* = 1.9; *SD* = 0.3	
Religiosity	How religious would you describe yourself as?	Five-point semantic differential scale	Not religious at all–very religious	*M* = 2.1; *SD* = 2.0	
Political attitude	How would you classify your own political views?	Five-point semantic differential scale	Very liberal–very conservative	*M* = 2.9; *SD* = 1.6	

*Note.N* = 105.

We used standardized content analysis to examine the screen recordings of the eye tracker and online searches with participants’ fixations to capture and evaluate the perceived content.^44,45^ Eye tracking provides valid information on whether certain textual, visual, and interactive elements of a webpage, social media post, or video are visually attended to, in what order, and for how long.^59–61^ The advantage of eye tracking over normal screen recording in relation to our study is that we can check exactly that a video was watched, that we can capture gaze patterns on web pages, and that we can capture which search results were viewed on search engine pages. Three trained coders coded the screen recordings with integrated eye tracks (based on Kessler & Guenther and Kessler & Zillich) for the procedural distribution of attention, perception patterns, navigation, and exploration paths on websites and search result pages, perceived content and issues, and selection on search engine pages and websites (see Table 4 for constructs/variables of the content analysis of the screen recordings, description, measurement details, frequencies, and means).^44,45^ The coding achieved satisfactory intercoder reliability (see Online Appendix Table 1 for the intercoder reliability values of the content analysis of the screen recordings according to Holsti and Cohen).^62,63^

**Table 4.** Constructs/variables of the content analysis of the screen recordings, description, measurement details, frequencies, and means.

Name	Description	Measurement details	Measurement items & characteristics	*Frequencies n (%)*	*Sample size (N)*	*Mean and standard deviation*
Task	Assigned video and search task	1–3	1 = Stricker 2 = Clemens Arvay 3 = Bill Gates	1 = 33 (32.0) 2 = 36 (35.0) 3 = 34 (33.0)	106	
Length of online behavior	Total length of online behavior	0–*x*	In seconds		106	*M* = 1005.4; *SD* = 435.5
Internet pages visited	Number of internet pages visited	0–*x*	Numeric value		1749	*M* = 16.9; *SD* = 9.7
Category of internet page	Category of the visited internet page	1–30	1 = Dictionary 2 = Search engine page 3 = Websites of ministries/authorities 6 = Online presence of a print product (newspaper) 7 = Online presence of a TV product 10 = Social media sites (excl. YouTube) 11 = Associations/networks/NGOs 12 = Website of the FOPH [BAG] 13 = Website of Stricker.tv 16 = Alternative media websites 17 = YouTube 18 = Fact-checking websites 20 = Website of Thomas Schauffert 21 = University 22 = Scientific journal 30 = Other (open)	1 = 86 (5.0) 2 = 671 (38.9) 3 = 46 (2.7) 6 = 150 (8.7) 7 = 54 (3.1) 10 = 32 (1.9) 11 = 89 (5.2) 12 = 20 (1.2) 13 = 20 (1.2) 16 = 32 (1.9) 17 = 265 (15.4) 18 = 36 (2.1) 20 = 21 (1.2) 21 = 29 (1.7) 22 = 59 (3.4) 30 = 114 (6.7)	1749	
Number of search engine pages	Number of search engine pages perceived	0–*x*	Numeric value		106	*M* = 6.7; *SD* = 6.1
Time with search engine	Total time spent on search engine internet pages	0–*x*	In seconds		106	*M* = 146.7; *SD* = 120.9
Fixation sequence on search engine internet pages	Sequence of fixations on search engine internet pages	1–4	1 = strictly linear (every single result is looked at; starting with the first result) 2 = linear (results are viewed from top continuously down with omissions) 3 = linear with a step back 4 = nonlinear (e.g., several jumps forward or backward in the course of gaze)	1 = 261 (50.8) 2 = 153 (29.8) 3 = 66 (12.8) 4 = 34 (6.6)	1749	
Vaccination reference search query	Vaccination reference of the typed search query	0–1	0 = no vaccination reference 1 = vaccination reference	0 = 402 (71.7) 1 = 159 (28.3)	580	*M* = 0.3; *SD* = 0.5
Search query length	Length of the search query	0–*x*	In a number of words		580	*M* = 3.4; *SD* = 1.9
Autocomplete search query	Autocomplete in the search query	0–1	0 = no 1 = yes	0 = 503 (89.7) 1 = 58 (10.3)	580	*M* = 0.1; *SD* = 0.3
Vaccination assessment Search query	Assessment of vaccination of the search query	0–3	0 = no assessment 1 = (somewhat) positive 2 = (somewhat) negative 3 = both positive and negative	0 = 80 (50.7) 1 = 9 (4.7) 2 = 65 (41.4) 3 = 5 (3.2)	159	
Number of search queries	Number of search queries in the search session	0–*x*	Numeric value		106	*M* = 5.8; *SD* = 4.8
Search engine result page	Search engine result page	1–*x*	1–*x*		1361	*M* = 1.0; *SD* = 0.2
Search engine result position	Position of the search engine result on the search engine page	1–*x*	1–*x*		1361	*M* = 3.7; *SD* = 2.6
Search engine result selection	Selection of search engine results	0–1	0 = not selected 1 = selected	0 = 867 (64.2) 1 = 483 (35.8)	1361	*M* = 0.4; *SD* = 0.5
Vaccination assessment search engine result	Assessment of vaccination on the search engine result	0–3	0 = no assessment 1 = (somewhat) positive 2 = (somewhat) negative 3 = both positive and negative	0 = 1184 (87.8) 1 = 58 (4.3) 2 = 91 (6.8) 3 = 15 (1.1)	1361	
Search engine results received	Number of search engine results received	0–*x*	Numeric value		106	*M* = 13.6; *SD* = 11.0
Search engine results selected	Number of selected search engine results	0–*x*	Numeric value		106	*M* = 4.9; *SD* = 3.3
Main topic of the website selected	Main topic of the website selected	1–*x*	1–*x* according to the topic list		980	
Vaccination assessment website	Assessment of vaccination on the website	0–3	0 = no assessment 1 = (somewhat) positive 2 = (somewhat) negative 3 = both positive and negative	0 = 629 (69.0) 1 = 169 (18.6) 2 = 89 (9.8) 3 = 24 (2.6)	980	
Misinformation (completely false) website	Presented misinformation (completely false) in the received website	0–1	0 = no misinformation (completely false) 1 = yes misinformation (completely false)	0 = 854 (92.3) 1 = 71 (7.7)	980	*M* = 0.1; *SD* = 0.3
Misinformation (half-truth) website	Presented misinformation (half-truth) in the received website	0–1	0 = no misinformation (half-truth) 1 = yes misinformation (half-truth)	0 = 845 (91.4) 1 = 80 (8.6)	980	*M* = 0.1; *SD* = 0.3
Fixation sequence websites	Sequence in which the participant fixates on the paragraphs of the website	1–4	1 = strictly linear 2 = linear 3 = linear with step back 4 = nonlinear	1 = 285 (41.2) 2 = 294 (42.5) 3 = 23 (3.3) 4 = 90 (13.0)	980	
Reception time website	Time spent on website reception	0–*x*	In seconds		980	*M* = 73.3; *SD* = 105.5
Received websites	Number of received websites	0–*x*	Numeric value		980	*M* = 9.5; *SD* = 5.1
Received provaccination websites	Number of received websites that are provaccination	0–x	Numeric value		106	*M* = 1.7; *SD* = 1.8
Received antivaccination websites	Number of received websites that are antivaccination	0–*x*	Numeric value		106	*M* = 0.8; *SD* = 1.1
Reception time provaccination websites	Reception time of received websites that are provaccination	0–*x*	In seconds		106	*M* = 196.7; *SD* = 258.9
Time received antivaccination websites	Reception time of received websites that are antivaccination	0–*x*	In seconds		106	*M* = 59.7; *SD* = 99.4
Total time on websites	Total time spent on perceiving all websites	0–*x*	In seconds		106	*M* = 694.5; *SD* = 385.9

### Sample description

One hundred and five subjects participated in the study; 58% were women (*n* = 61) and 41% were men (*n* = 43). The educational level was similar to the distribution in the Swiss population: compulsory school = 21% (*n* = 22), secondary education = 50% (*n* = 52); tertiary education = 30% (*n* = 36). The mean age was 36.4 years (*SD* = 17.4; min = 18 years; max = 80 years). Respondents considered themselves to be rather nonreligious and neither very liberal nor very conservative. Ninety-one people (87%) had been vaccinated against COVID-19, and 14 had not. The main reasons for not being vaccinated were fear of side effects and lack of belief in efficacy. According to self-reports, 10% of participants had already contracted COVID-19. The course of the disease was mild in all cases. Eighty-five people (83%) had already had a case of COVID-19 in the family or among acquaintances. Thirteen individuals reported belonging to the COVID-19 risk group. Online Appendix Table 2 reports the characteristics of the participants.

Self-perceived internet search skills were rather high before the stimulus and online search task (*M* = 1.9; five-point scale). The average frequency of internet searches reported was between hourly and daily. Respondents relatively frequently used YouTube to search for information (*M* = 3.2; five-point scale of 1–5); however, they used it more frequently for entertainment (*M* = 4.1). Information about the COVID-19 crisis on YouTube was generally somewhat more likely to be rated as understandable and helpful but also exaggerated and somewhat less likely to be rated as well researched, independent, trustworthy, or accurate (see Table 1). With regard to COVID-19, respondents generally trusted scientists, researchers, physicians, and medical staff more than politicians and relatives, acquaintances, or colleagues (see Table 3). Regarding their knowledge about vaccination, respondents were self-confident and, on average, agreed with the following statement “I know a lot about vaccination against the coronavirus.” In terms of self-assessed internet skills, self-confidence was also evident after the online search task. Respondents agreed most strongly with the following statement: “Personally, I find it easy to distinguish between what is true and what is false when I am reading up on current news” and least agreed with the statement that “I should take a course on how to find information online.” They reported encountering misinformation about COVID-19 in general and on the internet weekly, on average. Interestingly, they said they encountered misinformation less frequently on YouTube. We also asked how they respond when coming across news or information about COVID-19 vaccination that they suspected to be false. The highest levels of agreement were for “I ignore this information” and “I am annoyed by such information” and the lowest levels for “I point out to others that this information may be wrong” (for all mean values, including standard deviations, see Tables 1 to 3).

## Result

### Attitudes toward COVID-19 vaccination pre-video, post-video, and post-internet search

**Figure 2.**
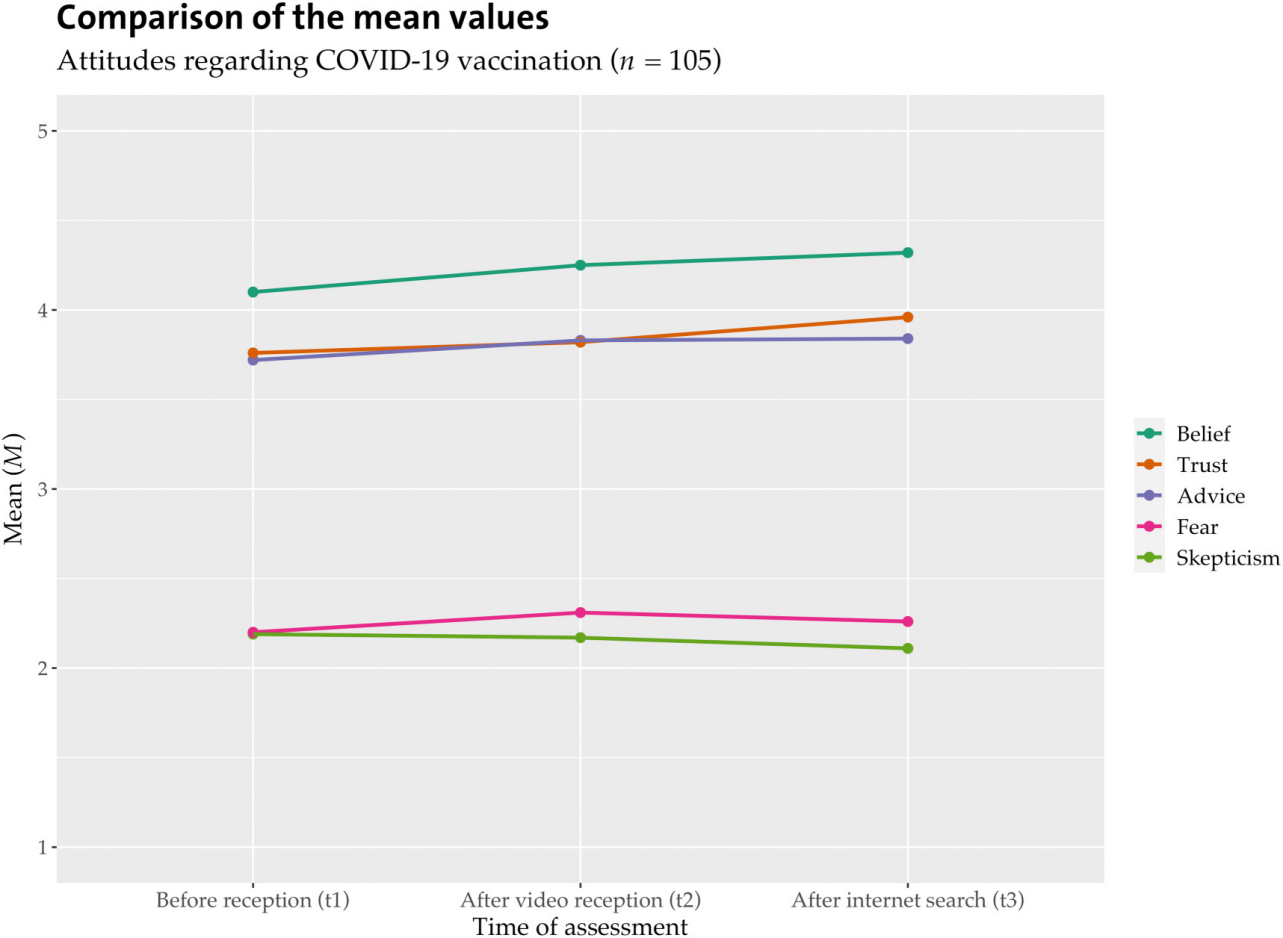
Attitudes in the total sample according to time of measurement.

Table 5 shows the mean values of the attitude items in the total sample and their change depending on the time of the query. For each item, a repeated-measures analysis of variance (ANOVA) was calculated for the entire sample. The three video types did not significantly differ in their (between-subjects) effects. Significant effects were found for the attitude items of belief and trust but not skepticism, the emotion of fear, or the behavior of giving advice. ANOVA with repeated measures on belief in vaccination efficacy (sphericity not assumed without correction: Mauchly-*W*(2) = .668, *p* < .001, Huynh-Feldt correction applied, *HF* = .887) shows that video reception and internet searches on COVID-19 vaccination influence belief (*F*(1,519,157,946) = 4.007, *p* < .030, η_p_^2^ = .037, *n* = 105). Bonferroni-corrected pairwise comparisons show that belief is significantly higher after internet research (*M* = 4.32, *SD* = 0.966) than before video reception (*M* = 4.10, *SD* = 1.165). The effect size *f* according to Cohen is .196 (a weak effect).^63^ ANOVA with repeated measures in terms of the trust (assumed sphericity: Mauchly-*W*(2) = .953, *p* = .085) shows that video reception and internet searches on COVID-19 vaccination have an effect on trust in vaccine efficacy (*F*(2,208) = 3.086, *p* = .048, η_p_^2^ = .029, *n* = 105). Bonferroni-corrected pairwise comparisons show no significant difference between all three measurement time points. Belief and trust in the efficacy of COVID-19 vaccination slightly increased after the reception of the misinforming video and slightly after internet research (see Figure 2).

**Table 5.** Mean values of the attitude items in the total sample according to time of measurement.

	Before video reception (t1)	After video reception (t2)	After internet search (t3)
Belief	4.10 (1.17)	4.25 (1.14)	4.32 (0.97)
Advice	3.75 (1.54)	3.85 (1.51)	3.84 (1.53)
Skepticism	2.19 (1.22)	2.17 (1.30)	2.10 (1.31)
Trust	3.76 (1.28)	3.82 (1.28)	3.96 (1.18)
Fear	2.20 (1.31)	2.31 (1.32)	2.27 (1.31)

*Note. M* (*SD*); five-point Likert scale: 1 = *do not agree at all* to 5 = *agree at all.*

To calculate the influence of prior attitude on the attitudes after the video reception and after the internet search, we calculated regression analysis regarding the dependent variables belief in the efficacy of the vaccine at timepoint 2 and timepoint 3 with the independent variable of prior attitude. This independent variable was an average index of the five attitude items at timepoint 1. Significant influences of prior attitude on attitudes after video reception (*F*(1,103) = 102.9, *p* < .001, *n* = 105) and on attitudes after internet search (*F*(1,102) = 136.9, *p* < .001, *n* = 104) were detected. Half of the dispersion of belief after video reception (50%) and after Internet research (57%) is explained by the independent variable of prior attitude.

To calculate the influence of prior attitude on the effect of video reception and internet search, we divided the sample into two groups using the average index of the five attitude variables; *M* < 3 = group with more negative attitudes; *M* > 4 = group with more positive attitudes). In each case, ANOVAs with repeated measures were calculated for the groups with the more positive (*n* = 73) and more negative (*n* = 14) attitudes. No significant results were found. From the direction of the mean values, however, it can be concluded that there are no differences between people with rather positive and rather negative attitudes. Belief in the efficacy of vaccination increased after video reception and after an internet search for those with more positive attitudes (*M*(t1) = 4.64, *SD* = 0.64; *M*(t2) = 4.75, *SD* = 0.62; *M*(t3) = 4.76, *SD* = 0.43) and those with more negative attitudes (*M*(t1) = 2.29, *SD* = 0.91; *M*(t2) = 2.36, *SD* = 1.22; *M*(t3) = 2.79, *SD* = 1.25). H1b receives no confirmation.

**Figure 3.**
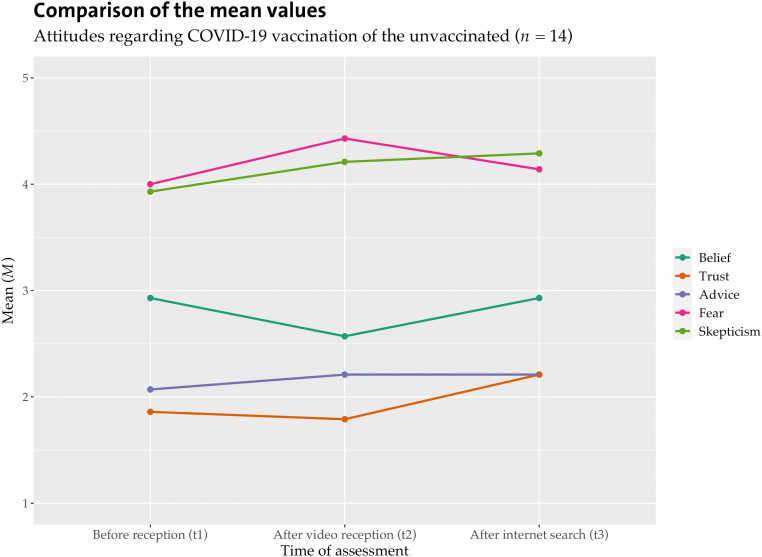
Attitudes of unvaccinated participants by the time of measurement.

Hypothesis 1a assumes that vaccination status has an impact on the effect of the video. The sample had 14 unvaccinated individuals. Because of the small number, the following findings must be considered with caution, but evidence for a differential effect does appear. Figures 2 and 3 show the mean values for the total sample versus the unvaccinated participants.

Vaccinated individuals show very similar effects as in the overall sample. The belief in the effectiveness of the vaccination increases. A repeated-measures ANOVA shows that video reception and internet searches for COVID-19 vaccination influence vaccinated individuals’ belief about vaccine efficacy (*F*(1,89) = 6.004, *p* < .016, *n* = 90). Bonferroni-corrected pairwise comparisons show that belief is significantly higher after internet research (*M* = 4.53, *SD* = 0.71) than before video reception (*M* = 4.28, *SD* = 1.06).

For the unvaccinated, the result looked somewhat different. A repeated-measures ANOVA shows that video reception and internet searches for COVID-19 vaccination influence unvaccinated individuals’ beliefs about vaccine efficacy (*F*(2,26) = 3.736, *p* = .037, η_p_^2^ = .223, *n* = 14). The Cohen effect size *f* is .536 (a strong effect).^63^ A repeated-measures ANOVA shows that video reception and internet searches for COVID-19 vaccination also have an effect on unvaccinated individuals’ trust in vaccine efficacy (*F*(1,911,198,692) = 3.086, *p* = .048, η_p_^2^ = .029, *n* = 14). The Cohen effect size *f* is .173 (a weak effect).^63^ Descriptive trends show that unvaccinated participants were even more skeptical after watching the video and even less likely to believe in and trust vaccine efficacy. After the internet search, belief returned to the initial level and trust increased, but skepticism also increased. The level of fear of vaccine side effects was higher than that of vaccinated participants and increased even more after the video reception. The internet search resulted in lower fear and more trust for those participants. Based on these findings, we conclude that internet search can improve trust and reduce fear but not decrease skepticism. H1a can be supported: The effect of the misinforming videos differs depending on recipients’ vaccination status.

In summary, receiving a misinforming video tended to have negative effects mostly for unvaccinated participants. Internet search led to positive results regardless of vaccination status or prior attitudes.

### Online information search and selective exposure

We asked directly after the video reception if participants would like to find out more about its content on the internet. Half of the respondents reported at least a strong to moderate need (51%); 77% stated that their internet search behavior in the study matched their normal/everyday online search behavior. Nearly all (96%) of participants stated that they had already informed themselves about COVID-19 vaccination on the internet before this study. Almost 70% of participants even stated that they do this frequently to very frequently; 65% of participants added that they had searched for specific content from vaccination critics on the internet at least once. After the search, most participants indicated that they were not able to confirm the video content they saw by means of their internet search.

The internet search behavior after video reception took an average of 17 minutes per person (for all mean values, including standard deviations, see Table 4), with a minimum of 4 and a maximum of 36 minutes. On average, 17 internet pages were opened (min. = 2; max. = 51). These were mostly search engine pages (39%), YouTube pages (15%), webpages of public print or TV products (12%) of associations/networks/NGOs (5%), online encyclopedias (5%), webpages of government entities (4%), scientific journals (3%), and fact-checking websites (2%). The high number of YouTube pages here can be explained by the study design. Since the corresponding YouTube video is set as the start page of the search, many participants started their information search directly on YouTube before switching to a search engine and from there to other websites. However, opening a website does not necessarily mean users consume the content on that page. Therefore, the websites received where the content was viewed are more relevant for analysis.

On average, 2.5 minutes were spent on a search engine page. The viewing behavior was mostly strictly linear in over a half of the cases (recipients looked at the search results strictly in order from top to bottom until they clicked on a result). In 30% of the cases, a result was skipped in the sequence, and in 13% of the cases, there was a look back to a previous result in the sequence after linear viewing behavior, which was then clicked on. The average of six search queries per session was entered in words. An average of 3.4 words was entered. Not using one-word searches hints at a more targeted search. Only 10% of the queries used Google's autocomplete function.

About one-third of the search queries typed in had an explicit reference to the topic of COVID-19 vaccination. Of these, half were without an explicit assessment, but over 40% had a negative assessment of the vaccination because, for example, death rates and vaccination risks and side effects were explicitly searched for. No correlation between prior attitude and valence of typed vaccine-related search questions could be detected. Search results were clicked on the first hit list page of a results page in almost all cases. On average, 14 search results were viewed on a results page, and participants clicked on five. The average position of a clicked search result was between three and four, with the most frequently clicked being position one (26%), the second position two (17%), the third position three (13%), and so on. This selection behavior corresponds to the normal selection behavior of internet users on search engine result pages in general. Twenty-eight percent of the queries had an explicit reference to vaccination; of the 1361 received search result teasers, an explicit vaccination assessment appeared in 12%.

The most frequently received website domains were youtube.org (10%), wikipedia.org (online encyclopedia; 3.5%), correctiv.org (German-language fact-checking website; 1.7%), bag-coronavirus.ch (web presence of the Swiss Federal Office of Public Health; 1.5%), medrxiv.org (preprint server for health sciences; 1.5%), news-medical.net (open-access medical and life science hub; 1.5), 20min.ch (web presence of a Swiss tabloid newspaper; 1.2%), stricker.tv (website of an anti-vaccination person present in one of the videos; 1.0%), thomasschauffert.com (website of an anti-vaccination person shown in one of the videos; 1.0%). The selected websites were diverse: Participants received 260 different website domains (URL domains). The most frequent topics were Bill Gates and the Bill and Melinda Gates Foundation, mRNA vaccine, Daniel Stricker and Clemens Arvay (anti-vaccination activists), vaccination side effects, and COVID-19 vaccine in general (see Online Appendix Table 3 for the main topics of the websites selected). The Bill and Melinda Gates Foundation, Daniel Stricker, and Clemens Arvay were specific content or protagonists in the misinforming videos. The website topics reflect the subjects of the stimulus videos. Although the videos were explicitly about vaccination, participants also frequently searched for specific information about their actors/sources. The vaccination assessment on the 980 received websites was positive in 19%, negative in 10%, and ambivalent in 3% of cases; 69% of the websites, however, had no explicit vaccination assessment at all. Approximately 8% of the websites contained explicit completely false misinformation, and 8% contained half-truths (often the same websites). All in all, the search behavior shows that misinforming content was selected and received relatively rarely. The gaze progression on the webpages was strictly linear (from top to bottom) without jumps in 41% of cases and linear with jumps in 43%, indicating intensive reading behavior and not just superficial scanning of content. On average, 10 webpages were received in a search session, of which two were pro-vaccination and one anti-vaccination. A website was received on average 1.5 minutes (max. 20 minutes). About 12 minutes were spent receiving webpages per search session, with about 3 minutes spent with explicit pro-vaccine websites and 1 minute with anti-vaccine websites. Thus, although the videos showed misinformation against vaccination and the vaccination-related search queries were rather negative, participants were more likely to receive more supportive and especially neutral information than (more) information against vaccination.

To test H2 on whether participants are more likely to search for and perceive attitude-consonant than dissonant content in their online search, we examined the effect of the prior attitude items on the valence of typed vaccine-related search questions, on the number of pro- and anti-vaccination webpages received and on the respective duration of reception as well as the effect on misinforming content received. Significance was not found in any of the calculated regressions. H2 therefore cannot be confirmed. Furthermore, no attitudinal variable correlated with the length of the online search. H3 can also not be confirmed.

### Credibility attribution to the misinforming videos

We also measured the credibility attributed to the speaker and the content of the video after reception and after the internet search. We found that internet searches significantly lowered the credibility initially attributed to both. Table 6 shows the differences in the mean values of the items. ANOVA with repeated measures of credibility attributed to the speaker/main actor of the respective video shows that the internet searches yielded significant effects (*F*(1,5889) = 21.202, *p* < .001, η_p_^2^ = .171, *n* = 104). The effect size *f* according to Cohen was .454, (a strong effect).^63^ ANOVA with repeated measures on credibility attributed to the video content shows that the internet searches also influence these (*F*(1,3769) = 15.387, *p* < .001, η_p_^2^ = .130, *n* = 104). The effect size *f* according to Cohen is .387 (a medium to strong effect).^63^ Thus, internet searches led to a decrease in the attributed credibility of the misinformation videos; this finding supports H4.

**Table 6.** Attributed credibility by the time of measurement.

	After video reception (t2)	After internet search (t3)
**Credibility content (index)**	2.2 (0.9)	1.9 (0.9)
Biased–unbiased	2.2 (1.1)	1.9 (1.2)
Unbelievable–believable	2.5 (1.2)	2.1 (1.2)
Trustworthy–not trustworthy	3.8 (1.1)	4.2 (1.0)
Inaccurate–accurate	2.4 (1.1)	2.0 (1.2)
Incomplete–complete	1.9 (1.0)	1.8 (1.1)
**Credibility speaker (index)**	2.3 (0.9)	1.9 (1.0)
Can be trusted–cannot be trusted	3.8 (1.1)	4.1 (1.2)
Fair–unfair	3.2 (1.0)	3.7 (1.0)
Inaccurate–accurate	2.3 (1.1)	2.0 (1.1)
Complete (tells the whole story)–incomplete (does not tell the whole story)	4.1 (1.1)	4.3 (0.9)
Unbiased–biased	4.1 (1.1)	4.3 (1.0)

*Note.M* (*SD*); five-point semantic differential scale.

## Discussion

In this observational and survey study, we investigated (a) the effect of misinforming YouTube videos about COVID-19 vaccination on attitudes toward vaccination and (b) the effect of subsequent online information searches on recipients’ attitudes and credibility attributions.

Misinforming videos were found to primarily affect the cognitive component of attitudes. Research on media effects on health communication has shown that cognitive rather than behavioral components of attitudes are influenced.^64^ We found in the study sample, which was generally more positive about COVID-19 vaccination, that the videos reinforced belief and trust in vaccine efficacy. The theory of cognitive dissonance offers a possible explanation.^28^ It can be assumed that video reception made the positive attitudes in our sample more salient. In other words, opposing arguments reinforced the positive attitudes. The internet search, which corrected the misinformation in the video, then further strengthened and confirmed the positive attitudes. Rather than misinformation causing doubt that recipients’ position might be wrong, it motivated them to defend existing positive attitudes via information seeking, also known as “defense-motivated information processing.”^65,66^ As Taddicken and Wolff found for climate change misinformation and subsequent online search, striving for confirmation is a typical behavior when coping with dissonant content.^54^ Confirmation bias is often considered problematic in terms of biases among polarized users. However, it can be interpreted as positive in this case because the “users are not persuaded by misinformation because of their confirmation-seeking behavior, thus leading to disinformation resistance” (p. 18).^54^ Our study indicates different types of misinformation effects. Most notably, it shows rejection of misinformation and salientization of the perception of one's own prior attitudes.^67^

The positive effects of the search are thus comparable to effective debunking texts, in which misinformation is first presented and then debunked.^31,34,56^ Furthermore, internet searches led to a decrease in the videos’ attributed credibility. The impact of information can depend significantly on ascribed credibility, so subsequent information search seems to be an effective way to counteract the negative effects of misinformation.^34^

The results of the study also indicate that the effects of the video are especially negative in unvaccinated individuals. Their negative preconceptions are intensified by the attitude-consistent arguments in the videos, whereas vaccinated individuals are more likely to disregard the dissonant content. However, the internet search also led to more positive attitudes toward COVID-19 vaccination among the unvaccinated individuals than after video reception. This contradicts the selective exposure approach but is logical in that users are habitualized in their internet search and look attitude-independently for information that is mostly neutral.^45^

Only half of the respondents stated that they had a strong to moderate need to find out more about the content after watching the video. A measure in the fight against misinformation could start here and motivate citizens to search for the correctness of YouTube content after viewing it. Furthermore, most users simply ignore misinformation they encounter and do not actively point it out. Here, too, motivation and even simplification of the reporting process on platforms could counteract the spread of misinformation. From our own experience, that reporting process is unnecessarily cumbersome and complicated and rarely successful, reducing the motivation to report. Increasing motivation to actively seek online information after the occurrence of misinformation through targeted interventions is also particularly important because a recent online survey study by Kim et al.^68^ shows that the exposure to misinformation deters subsequent online information seeking, implying that misinformation is associated with lower levels of information insufficiency. Those exposed to misinformation may feel that they already have all the information they need and do not need to search further, which leads to greater information avoidance and less systematic processing of additional COVID-19 information.

We examined how participants’ internet searches appeared. With regard to the general selection and gaze behavior on search engine pages and websites, the search behaviors corresponded to the normal internet search for health information, which has also been observed in other eye-tracking studies.^45^ The input of several search words and the (strictly) linear reception of webpages is more evidence of an in-depth, targeted, and systematic search for information than of a superficial scanning of information.^69^ Of course, this behavior may be a consequence of the methodology and task of this study. Regarding the visited websites, Wikipedia played a relatively important role, as is usual for health topics.^69^ The WHO website was not among the most visited. However, fact-checking websites and governmental websites with validated information were used more frequently than websites of vaccination opponents. It can be assumed that some subjects first became aware of certain conspiracy theorists through the videos and therefore took a closer look at their websites, even if they did not believe them. Although respondents generally trust scientists and researchers, as well as physicians and medical personnel, regarding COVID-19, relatively few scientific websites were accessed. This may be because of poor discoverability or because recipients feared that the content was too scientific and could not be understood. Further research and qualitative thinking-aloud interviews are necessary to better understand the reasons users chose specific websites.

Overall, search behavior shows that misinforming content was selected and perceived relatively rarely. This is despite the fact that the typed search queries with reference to the COVID-19 vaccination were often also negative and with reference to the misinformation from the video. The fact that one searches for something negative does not mean that one gets negative search results or web pages from a search engine. The valence of the search words entered and the search duration was independent of the participants’ prior attitudes. In general, participants were more likely to perceive supportive and mostly neutral information about vaccination rather than for (more) information against vaccination, independent of their prior attitudes. Previous studies on internet search behavior also indicate that it appears to be independent of prior attitudes and relatively stable and habitualized with respect to the search, selection, and reception of health information.^45^ These findings contradict an assumed selective exposure effect, which has been demonstrated in some studies with specific, more artificial experimental designs.^49,51^ However, research has established that with unlimited reception time for the actual World Wide Web, users turn to both attitudinal consonant and dissonant content.^45,52^ This finding also partly contradicts the filter bubble thesis.^70–72^

### Limitation

The sample is relatively large for an eye-tracking study, considering that at least 1 hour of time per subject is spent in the laboratory.^73^ However, it would be desirable to have a larger sample to capture smaller effects (below *f* < .2) and the effects of important influencing variables on the detected effects. Particularly for the effects observed in unvaccinated participants, a larger sample would be needed to support the findings.

Participants in the study were actively encouraged to search for relevant information. This behavior was enforced in the study and might not occur or occur to this extent in a normal situation after the video repetition.^45^ Such systematic searching as recorded in the study may be less common outside the laboratory. Eye tracking in the laboratory is an obstructive method. Participants are aware that they are being observed, with the risk that they will behave in a socially desirable manner rather different from the one they would show if they were unobserved. In the questionnaires assessing attitudes toward COVID-19 vaccination, the same questions were asked three times in the same way. The obvious dependent variable could also cause respondents to behave in socially desirable or more defensive rather than natural ways. Also critical to the generalizability of the study results is that only three videos were tested. Misinformation videos on COVID-19 vaccination can look very different and accordingly, videos that look very different could have stronger or weaker effects.^55^

## Conclusion

Misinformation on social media (e.g. on the video platform YouTube) is a major barrier to collective action in a health crisis. Misinformed citizens are less likely to take action to mitigate the COVID-19 pandemic or to get vaccinated. Our objective was to examine the impact of YouTube misinformation and gain insights into individual verification strategies via information seeking. We found that misinforming videos about the COVID-19 vaccination can have a negative effect on unvaccinated citizens in particular. Exposure to such videos decreased their belief and trust in the vaccine. Especially for this vulnerable group, it is important to take action against misinformation on YouTube.

However, a subsequent internet search generally yielded positive results, regardless of vaccination status. This suggests that it is important to motivate users to verify online content through further searching on their own. Other studies also underline the need for policymakers and health professionals to encourage and assist the general public to search for reliable information about COVID-19 measures. Doing so can be an important step to stimulate citizens’ adoption of preventive behaviors. Internet search motivation interventions can be done, for example, through targeted information campaigns on search engines and websites or banners and notices on social media platforms. Science and health literacy campaigns aimed at promoting people's verification and correction of misinformation need to emphasize the threat that it poses to others and our shared responsibility to identify it, which motivates users to do their part to verify and correct it.

## Supplemental Material

sj-docx-1-dhj-10.1177_20552076231177131 - Supplemental material for COVID-19 misinformation on YouTube: An analysis of its impact and subsequent online information searches for verification Click here for additional data file. Supplemental material, sj-docx-1-dhj-10.1177_20552076231177131 for COVID-19 misinformation on YouTube: An analysis of its impact and subsequent online information searches for verification by Sabrina Heike Kessler and Edda Humprecht in DIGITAL HEALTH
