# Supplementary material for: COVID-19 misinformation on YouTube: An analysis of its impact and subsequent online information searches for verification
Source: Digit Health. 2023 May 25;9:20552076231177131. doi: 10.1177/20552076231177131 (PMC10226045; doi:10.1177/20552076231177131)
Supplement: sj-docx-1-dhj-10.1177_20552076231177131 - Supplemental material for COVID-19 misinformation on YouTube: An analysis of its impact and subsequent online information searches for verification [file sj-docx-1-dhj-10.1177_20552076231177131.docx]

**Appendix**

Table 1: Intercoder reliability values of the content analysis of the screen recordings according to Holsti (1969) and Cohen (1988)

| Variable name | Codes | Holsti | Cohen’s Kappa |
| --- | --- | --- | --- |
| Coder | Two coders | 0 | 0 |
| Test person | 1–105 | 1 | 1 |
| Task | 1 = Stricker  2 = Clemens Arvay  3 = Bill Gates | 1 | 1 |
| File name | nominal | 1 | 1 |
| Length of online behavior | 1–x in seconds | 1 | 1 |
| Internet page visited | string |  |  |
| Category internet page | 1–30 | .89 | .84 |
| Number of search engine pages | 1–x | .70 | .63 |
| Time with search engine | 1–x in seconds | .70^a^ | .68^a^ |
| Fixation sequence on search engine internet pages | 1 = strictly linear  2 = linear  3 = linear with step back  4 = nonlinear | .74 | .58 |
| Search query | string |  |  |
| Vaccination reference search query | 0 = no vaccination reference  1 = vaccination reference | 1 | 1 |
| Search query length | 1–x | .94 | .93 |
| Autocomplete search query | 0 = no  1 = yes | .97 | .87 |
| Number of search queries | 1–x | 1 | 1 |
| Search engine result page | 1–x | 1 | 1 |
| Search engine result position | 1–x | .75 | .69 |
| Search engine result selection | 0 = not selected  1 = selected | .90 | .78 |
| Vaccination assessment search engine result | 0 = no assessment  1 = (somewhat) positive  2 = (somewhat) negative  3 = both positive and negative | .81 | .59 |
| Search engine results received | 1–x | .56 | .53 |
| Search engine results selected | 1–x | .78 | .73 |
| Website | string |  |  |
| Internet page/domain | string | .87 | .87 |
| Main topic of the website selected | 1–x according to topic list | .70 | .67 |
| Vaccination assessment website | 0 = no assessment  1 = (somewhat) positive  2 = (somewhat) negative  3 = both positive and negative | .84 | .76 |
| Misinformation (completely false) | 0 = no misinformation (completely false)  1 = yes misinformation (completely false) | .91 | .51 |
| Misinformation (half-truth) | 0 = no misinformation (half-truth)  1 = yes misinformation (half-truth) | .87 | .59 |
| Website fixation sequence | 1 = strictly linear  2 = linear  3 = linear with step back  4 = nonlinear | .80 | .74 |
| Website reception time | 1–x in seconds | .82^a^ | .81^a^ |

Note: ^a^tolerance +/- 5 sec

Table 2: Characteristics of the participants

| Variables | Frequencies *n* (%) | *M* & *SD* |
| --- | --- | --- |
| Gender | female = 61 (58%);  male = 43 (41%);  diverse = 1 (1%) |  |
| Education | compulsory school = 22 (21%),  secondary education = 52 (50%);  tertiary education = 36 (30%) |  |
| Age | 18–x | *M* = 36.4; *SD* = 17.4 |
| Religiosity | not religious at all–very religious (5-point-scale) | *M* = 2.1; *SD* = 2.0 |
| Political attitude | very liberal–very conservative (5-point-scale) | *M* = 2.9; *SD* = 1.6 |
| COVID-19 vaccination | yes = 91 (87%);  no = 14 (13%) |  |
| Personal COVID-19 disease | yes = 10 (10%);  no = 95 (90%) |  |
| COVID-19 disease in the family or among acquaintances | yes = 87 (83%);  no = 16 (17%) |  |
| COVID-19 risk group | yes = 13 (12%)  no = 102 (88%) |  |

Table 3: Main topics of the websites selected

| Code | Topic | *Frequencies n (%)*  Main topic of the websites selected^b^ |
| --- | --- | --- |
| 1 | Bill Gates + Bill & Melinda Gates Foundation^A^ | 112 (13.9) |
| 2 | mRNA vaccine | 99 (12.3) |
| 3 | Daniel Stricker^A^ | 92 (11.4) |
| 4 | Clemens Arvay^A^ | 85 (10.5) |
| 5 | Vaccination side effects | 72 (8.9) |
| 6 | COVID vaccine | 65 (8.1) |
| 7 | Death of father in hospital^A^ | 46 (5.7) |
| 8 | Thomas Schauffert^A^ | 36 (4.5) |
| 9 | Conspiracy theories in general | 31 (3.8) |
| 10 | Opponents of measures | 23 (2.9) |
| 11 | COVID-19 pandemic | 22 (2.7) |
| 12 | Thorsten Schulte^A^ | 22 (2.7) |
| 13 | Vaccinations in general (not specifically COVID-19) | 18 (2.2) |
| 14 | Vaccination recommendations in general | 15 (1.9) |
| 15 | Daniel Gugger^A^ | 15 (1.9) |
| 16 | Development of COVID-19 diseases & deaths (national) | 13 (1.6) |
| 17 | COVID disease, course of the disease | 12 (1.5) |
| 18 | Fact-checking | 9 (1.1) |
| 19 | Corona Party Switzerland | 9 (1.1) |
| 30 | Other | 120 (13.2) |

Notes: sample size *N* = 980; ^A^ specific content or protagonists in the respective misinforming videos
